# American Medical Association

**Published:** 1853-07

**Authors:** 


*American Medical Association.*

At the meeting in New York, in May last, Dr. YANDELL, of Ky., presented a report from Dr. S. D. GROSS, on the result of surgical operations for the relief of malignant diseases, in which he arrives at the following conclusions:

*First*—That cancerous affections, particularly those of the mammary gland, have always, with a few rare exceptions, been regarded by practitioners as incurable by the knife and escharotics. This opinion, commencing with Hippocrates, the father of Medicine, has prevailed from the earliest records of the profession, to the present moment. Nature never cures a disease of the kind; nor can this be effected by any medicine, or internal remedies, known to the profession.

*Secondly*—That excision, however early and thoroughly executed, is nearly always, in genuine cancer, followed by relapse, at a period varying from a few weeks to several months, from the time of the operation.

*Thirdly*—That nearly all practitioners, from the time of Hippocrates to the present day, have been, and are still averse to any operation for the removal of cancerous tumor, after the establishment of ulceration, rapid growth, firm adhesion, organic change in the skin, lymphatic invasion, the cancerous dyscracy, or serious constitutional derangements; on the ground that, if had recourse to, under these circumstances, the malady almost inevitably recurs in a very short time, and frequently destroys the patient more rapidly than when it is permitted to pursue its own course.

*Fourthly*—That in all cases of *acute* carcinoma, or, in other words, in all cases of this disease, attended with very rapid development and great bulk of the tumor, extirpation is improper and unjustifiable, inasmuch as it will only tend to expedite the fatal result, which, under such circumstances, always takes place in a very short time.

*Fifthly*—That all operations performed for the removal of encephaloid cancer and its different varieties, are more certainly followed by rapid relapse than operations performed upon scirrhus or hard cancer.

*Sixthly*—That in nearly all the operations for cancerous diseases, hitherto reported, the history has been imperfectly presented, being deficient in the details which are necessary to a complete and thorough understanding of the subject in each case. This remark is particularly true in reference to the diagnosis of the malady, the minute examination of the morbid structure, and the history of the case after the operation, as to the period of relapse, the time and nature of the patient's death, and the result of the post-mortem examination.

*Seventhly*—That cancerous affections of the lip and skin, now usually described under the name of cancroid diseases, are less liable to relapse after extirpation than genuine cancerous maladies, or those which are characterized by the existence of the true cancer-cell and cancer-juice.

*Eighthly*—That, although practitioners have always been aware, from the earliest professional records, of the great liability of cancer to relapse after extirpation, a great majority of them have always been, and still are, in favor of operation in the early stage of the disease, especially in scirrhus, before the tumor has made much progress, before there is any disease of the lympatic ganglions, or any evidence of the cancerous cachexy.

*Ninthly*—That many cases of tumors, especially tumors of the breast and testicles, supposed to be cancerous, are in reality not cancerous, but of a benign character, and consequently, readily curable by ablation, whether effected by the knife or by escharotics. It is to this circumstance that we must attribute the astonishing success which is said to have attended the practice of Hill, of Scotland, Nooth, of England, and Flajani, of Italy.

*Tenthly*—That all operators insist upon the most thorough excision possible; removing, not merely the deceased mass, but also a portion of the surrounding and apparently healthy tissues, as well as all enlarged and indurated ganglions.

*Eleventhly*—That the practice has always prevailed, and still obtains, to save, if possible, a sufficient amount of healthy integument to cover the wound, and to unite, if possible, the wound by the first intention; on the ground that these precautions will tend much to retard, if not to prevent, a recurrence of the disease.

*Twelfthly*—That much stress is laid by writers upon a properly regulated diet, and attention to the bowels and secretions after operation, as means of retarding and preventing relapse.

*Thirteenthly*—That there is no remedy, medicine or method of treatment which has the power, so far as we are enabled to judge of its virtues, of preventing the reproduction of the morbid action after operation, no matter how early or how thoroughly it may be performed.

*Fourteenthly*—That life has occasionally been prolonged and even saved by operation after relapse, and in some of the remarkable cases mentioned in a previous part of this report; but that, as a general rule, such a procedure is as incompetent to effect a permanent cure as a first extirpation.

The following is the report of the Committee on Nominations:

The Committee on Nominations, in fulfilling the duty of their appointment, propose to continue most of the Special Committees appointed by the Association in May, 1851, and May, 1852, and to appoint several new Special Committees. They, therefore, submit the following list of Chairmen of Special Committees, with the subjects to them committed:

1. Dr. D. F. Condie, of Philadelphia, Penn., "On the causes of Tubercular Disease."
2. Dr. James Jones, of New-Orleans, La., "On the Mutual Relations of Yellow and Bilious Remittent Fever."
3. Dr. R. S. Holmes, of St. Louis, Mo., "On Epidemic Erysipelas."
4. Dr. Geo. B. Wood, of Philadelphia, Penn., "On Diseases of Parasitic Origin."
5. Dr. R. D. Arnold, of Savannah, Ga., "On the Physiological Peculiarities and Diseases of Negroes."

6. Dr. James R. Wood, of New York, "On Statistics of the Operation for the removal of Stone in the Bladder."

7. Dr. F. Peyre Porcher, of Charleston, S. C., "On the Toxicological and Medicinal Properties of our Cryptogamic Plants."

8. Dr. Goodrich A. Wilson, of Virginia, "On Cholera, and its Relations to Congestive Fever—their Analogy or Identity."

9. Dr. Worthington Hooker, of Connecticut, "On Epidemics of New England and New York."

10. Dr. John L. Atlee, of Lancaster, Peen., "On Epidemics of New Jersey, Pennsylvania, Delaware and Maryland."

11. Dr. D. J. Cain, of Charleston, S. C., "On Epidemics of South Carolina, Florida, Georgia and Alabama."

12. Dr. W. L. Sutton, of Georgetown, Ky., "On Epidemics Tennessee and Kentucky."

13. Dr. Thomas Reyburn, of St. Louis, Mo., "On Epidemics of Missouri, Illinois, Iowa and Wisconsin."

14. Dr. George Mendenhall, of Cincinnati. Ohio, "On Epidemics of Ohio, Indiana and Michigan."

15. Dr. E. D. Fenner, of New Orleans, La., "On Epidemics of Mississippi, Louisiana, Texas and Arkansas."

16. Dr. Chas. A. Lee, of New York, "On Domestic Hygiene."

17. Dr. Daniel Brainard, of Chicago, Ill., "On the Constitutional and Local Treatment of Carcinoma."

18. Dr. N. S. Davis, of Chicago, Ill., "On the Influence of Local Circumstances on the Origin and Prevalence of Typhoid Fever."

19. Dr. George Engelman, of St. Louis, Mo., "On the Influence of Geological Formation on the Character of Disease."

20. Dr. Henry M. Bullitt, of Louisville, Ky., "On the Use and Effect of Applications of Nitrate of Silver to the Throat, either in Local or General Disease."

21. Dr. Robert F. Campbell, of Augusta, Ga., "On the Pathogenic Influence of Feather Beds."

22. Dr. James Bolton, of Richmond, Va., "On the Administration of Anæsthetic Agents during Parturition."

23. Dr. Henry Taylor, of Mount Clemens, Michigan, "On Dysentery."

24. Dr. F. Donaldson, of Baltimore, Md., "On the Present and Prospective Value of the Microscope in Disease."

25. Dr. R. L. Howard, of Columbus, Ohio, "On the Pathology and Treatment of Scrofula."

*Committee on Plans of Organization for State and County Societies*—Isaac Hays, M. D., of Penn., Chairman; Worthington Hooker, M. D., of Connecticut; Josiah Andrews, M. D., of Mich.; B. R. Wellford, of Virginia; A. L. Pearson, M. D., of Mass.

*Committee on Medical Literature*—T. S. Bell, M. D., of Ky., Chairman; Samuel H. Pennington, M. D., of New Jersey; Ed.

H. Parker, M. D., of New Hampshire; William K. Bowling, M. D., of Tennessee; Zina Pitcher, M. D., of Mich.

*Committee on Medical Education*—B. R. Welford, M. D., of Virginia, Chairman; Resign Lowe, M. D., of Iowa; Lyndon A. Smith, M. D., of New Jersey; Jacob Bigelow, M. D., of Mass.; L. A. Dugas, M. D., of Georgia.

*Committee on Volunteer Communications*—Drs. C. A. Pope, Thos. Reyburn, John S. Moore, J. B. Johnson and A. Litton, of St. Louis, Mo.

*Committee of Arrangements*—Drs. J. R. Washington, J. S. Moore, S. Pollok, Thos. Reyburn, J. O'Farrar, W. M. McPheeters, C. W. Hempstead and E. S. Lemoine, of St. Louis, Mo.

*Committee on Publications*—Dr. D. F. Condie, Pennsylvania, Chairman; Dr. E. L. Beadle, of New York; Dr. A. Stille, Pennsylvania; Dr. I. Hays, Pennsylvania; Dr. E. S. Lemoine, of Missouri: Dr. G. Emerson, Pennsylvania; Dr. G. W. Norris, Pennsylvania.

On motion of Dr. WATSON, of New York, the name of the Committee on Volunteer Communications was changed to that of Committee on Prize Essays.

Dr. WELLFORD resigned his place as Chairman of Committee on Medical Education, and the President was authorized to fill the vacancy.

The vacancy was subsequently filled by the appointment of Dr. J. L. Cabell, of the University of Virginia.

